# Short-term sick leave and future risk of sickness absence and unemployment - the impact of health status

**DOI:** 10.1186/1471-2458-12-861

**Published:** 2012-10-10

**Authors:** Hanna Hultin, Christina Lindholm, Mauricio Malfert, Jette Möller

**Affiliations:** 1 Department of Public Health Sciences, Karolinska Institutet, Stockholm, Sweden; 2 Department of Clinical Neuroscience, Karolinska Institutet, Stockholm, Sweden; 3 Uppsala Clinical Research Center, Uppsala University Hospital, Uppsala, Sweden

**Keywords:** Short-term sick leave, Health status, Future sickness absence, Unemployment, Population-based study

## Abstract

**Background:**

In previous studies the authors have found sick leave to be a predictor of future sick leave, unemployment and disability pension. Although sick leave reflects underlying health problems, some studies have suggested that sick leave may have consequences beyond the consequences of the underlying illness. However, few studies have aimed at studying consequences of sick leave while adjusting for ill health. This study aims to explore whether short-term sick leave increases the risk of future long-term sick leave, disability pension, and unemployment. Furthermore, we aim to control for the potentially confounding effects of physical and mental health status.

**Methods:**

Data were gathered from the Stockholm Public Health Cohort (SPHC), restricted to 11,156 employed individuals (48.6% men) aged 18–59, without long-term sick leave, disability pension or in-patient care the year before inclusion (2002). These were followed-up with regard to unemployment, long-term sick leave, and disability pension in 2006 and 2007.

Odds ratios (OR) with corresponding 95% confidence intervals (CI) were estimated by logistic regression, controlling for six different measures of health status (limiting long-standing illness, self-rated health, mental health, somatic disease, musculoskeletal pain and in-patient care) and socio-demographic factors.

**Results:**

Results from the unadjusted analyses indicated increased risks of long-term sick leave (OR 2.00; CI 1.62-2.46) and short-term unemployment (OR 1.76; CI 1.35-2.29) for individuals exposed to more than one short-term sick-leave spell. There were no increased odds of long-term unemployment (OR 0.54; CI 0.28-1.04) or disability pension (OR 0.72; CI 0.42-1.24). After adjusting for the different measures of health status the odds ratio for short-term unemployment was not statistically significant (OR 1.29; CI 0.97-1.74). The odds ratios for the other outcomes slightly increased after adjustment for the used measures of health status.

**Conclusions:**

The results support the assumption that short-term sick leave may have consequences for future sick leave beyond the effect of ill health. The results point to the importance of paying attention to short-term sick leave in order to prevent subsequent sickness absence.

## Background

Sick leave is by definition absence from work due to illness, and sick leave is often used as an indicator of health [1-3]. In a number of studies, long-term sick leave has been shown to predict disability pension [4-17]. Studies of long-term sick leave have also indicated adverse financial and social effects, like a higher risk of unemployment and job termination [11,18,19]. A systematic review of factors associated with long-term sick leave concluded that there was only scientific evidence for an association with age and previous history of sick leave [20]. Additionally, age, sex, educational level, marital status, smoking, and adverse working conditions have been identified as risk factors for disability pension [5,7,12,21-28].

A few studies have focused on short-term sick leave as a predictor of long-term sick leave or disability pension; for instance, the number of days and spells of sick leave have been shown to predict later sick leave and disability pension [29-31]. Although sick leave often is assumed to have consequences beyond the consequences of the underlying ill health, few studies have attempted to study the possible effect of sick leave while trying to adjust for the illness that it reflects [32,33]. In 2004, seven studies of the consequences of sick leave were scrutinized in a systematic review, but none of the included studies had controlled for ill health [33]. In 2009, an attempt to separate the consequences of sick leave from those of ill health was done using self-reports on the consequences of sick leave, specifically requesting the respondent to separate these from the consequences of disease/illness [32]. Despite the attempts, the authors indicated that there was uncertainty regarding to what degree the consequences studied could purely be attributed to sick leave. The possibility to study the effect of sick leave independently of health status increases when high quality data on ill health, before and during sick leave, is available.

In the present study we aim to explore whether short-term sick leave (STSL) increases the risk of future long-term sick leave, disability pension, and unemployment. Furthermore, we aim to control for the potentially confounding effects of physical and mental health status.

## Methods

This is a prospective cohort study based on data from the Stockholm Public Health Cohort (SPHC). The SPHC is based on random samples of the population of Stockholm County aged 18 to 84 years who participated in the Stockholm Public Health Surveys in both 2002 and a follow-up in 2007. In 2002 a total of 31,182 individuals responded to the baseline postal questionnaire (response rate 62.4%) and in 2007 these were reassessed in a further health survey in which 23,794 participated (retention rate 76%). Information from Swedish health and administrative registers was linked using each citizen’s personal identity numbers.

At the time of this study, all employees in Sweden were covered by the same sickness-benefit insurance, which after one qualifying day covered up to 80% of the income below a given limit, for full- or part-time sick leave. The first 14 days were financed by the employer, and thereafter by the Swedish National Social Insurance Agency. After seven sick-leave days, a medical certificate was required [34,35].

The study has been approved by The Regional Ethical Review Board in Stockholm and conforms to the principles of the Helsinki Declaration. All participants of the Stockholm Public Health Cohort have given informed written consent.

### Study sample

For the purpose of this study we restricted the cohort to individuals (48.6% men) aged 18–59 in 2002. We excluded individuals who were unemployed, on full or part-time disability pension or on old-age pension in 2002. Furthermore, we excluded individuals who had more than 30 days sick-leave in 2001, or who had been admitted for in-patient care for more than three days or on more than three occasions in 2001. This resulted in final study sample of 11,156 individuals. Of the total 12,638 responders who were excluded from the study sample, 79.7% were due to either employment restrictions, age restrictions or a combination thereof.

### Exposure

Information regarding short-term sick leave was assessed through the questionnaire in 2002 using the two questions; “Have you been on sick leave during the last 12 months?” (with response alternatives as ‘No’, ’Yes, once’, ’Yes, 2–4 times’, ’Yes, 5 times or more’) and “How many days in total have you been on sick leave during the last 12 months?” (with response alternatives as ‘Not absent’, ‘1-7 days’, ‘8-30 days’, ‘31-90 days’, ‘More than 90 days’). Exposure to high STSL was defined as those stating one of the following combinations: ‘Yes, 2–4 times’ and ‘1-7 days’ in total; ‘Yes, 5 times or more’ and ‘1-7 days’ in total; or ‘Yes, 5 times or more’ and ‘8-30 days’ in total. Answering ‘Yes, once’ and ‘1-7 days’ was defined as 1 short-term sick-leave spell, low STSL. Non-exposure was defined as answering ‘No’ and ‘Not absent’ to the two questions. Other combinations of responses were not included in the analyses, leaving a study group of 9844 individuals. Characteristics of these individuals are presented in Table 1.

**Table 1:** Characteristics of the study group in 2002 (n=9844), n (%)

		**Short-term sick-leave (STSL) spells in 2002**			
		**No spell n (%)**	**Low STSL (1 spell) n (%)**	**High STSL (>1 spell) n (%)**	P-value*
	Total	4895 (49.7)	3125 (31.8)	1824 (18.5)	
Sex	Men	2733 (56.8)	1430 (29.3)	714 (14.6)	
	Women	2162 (43.5)	1695 (34.1)	1110 (22.4)	< 0.0001
Age	18-29	429 (33.0)	425 (32.7)	446 (34.3)	
	30-44	1828 (45.0)	1363 (33.6)	871 (21.4)	
	45-59	2638 (58.9)	1337 (29.8)	507 (11.3)	< 0.0001
Socio-economic position 2002	Manual and skilled manual worker	953 (44.8)	697 (32.8)	477 (22.4)	
	Low non-manual workers	554 (43.4)	447 (35.0)	277 (21.7)	
	Middle and high non-manual workers	2549 (48.4)	1748 (33.2)	968 (18.4)	
	Self-employed and farmers	741 (73.3)	189 (18.7)	81 (8.0)	< 0.0001
	Missing	98	44	21	
Born outside of Sweden	Yes	701 (55.1)	379 (29.8)	193 (15.2)	
	No	4179 (48.9)	2738 (32.1)	1626 (19.0)	< 0.0001
	Missing	15	8	5	
Self-rated health	Very good/Good	4275 (51.2)	2652 (31.8)	1418 (17.0)	
	Fair/Bad/Very bad	579 (41.1)	446 (31.6)	385 (27.3)	< 0.0001
	Missing	41	27	21	
Mental wellbeing measured with GHQ 12	Yes to < 3 items	4103 (51.9)	2485 (31.4)	1315 (16.6)	
	Yes to >= 3 items	715 (40.1)	592 (33.2)	477 (26.7)	< 0.0001
	Missing	77	48	32	
Limiting longstanding illness	Yes, to a high degree/Yes, to some degree	399 (43.6)	264 (28.8)	253 (27.6)	
	No	4459 (50.4)	2830 (32.0)	1555 (17.6)	< 0.0001
	Missing	37	31	16	

*P-values for Chi square tests.

### Outcomes

Four outcomes were assessed; long-term sick leave, disability pension and short- and long-term unemployment. Long-term sick leave was defined as having more than 30 sick-leave days during 2007 and disability pension as the occurrence of full or part time disability pension in 2007. Both long-term sick leave and disability pension was measured by register data from the Swedish National Social Insurance Agency. Unemployment was assessed based on the number of reimbursed days from the unemployment insurance fund according to the longitudinal integration database for health insurance and labor market studies during 2006 (2007 not available). Short-term unemployment was defined as having 1–180 reimbursed days and long-term unemployment defined as having more than 180 reimbursed days.

### Confounders

Information regarding potential socio-demographic and health-related confounders was retrieved from the 2002 survey and register data on country of birth and in-patient care in 2002. *Age* was categorized into three groups: 18–29, 30–44 and 45–59 years, and the youngest group were used as reference category. *Socio-economic status* (SES) was measured through a question on the respondent’s occupation and classified in accordance with Statistics Sweden classification [36]. For the analyses each participant was allocated to one of the following four socio-economic groups: Higher and intermediate non-manual employees, lower non-manual employees, manual workers (skilled and unskilled), and self-employed (self-employed and farmers). In the analyses the lower non-manual employees were used as the reference group. Other measures of socio-economic position, such as income and education, were also tested. *Country of birth* was based on a survey question, dichotomised as born in Sweden or elsewhere.

*Self-rated health* (SRH) was measured by one survey question with five response alternatives, varying from very bad to very good. Adverse self-rated health was defined as response alternatives; ‘very bad’, ‘bad’ and ‘fair’ [37,38]. *Limiting longstanding illness* (LLSI) was based on two questions; “Do you have a long-standing illness, ailment due to an accident, handicap or other weakness?” with response alternatives ‘yes’ or ‘no’ and if yes, the respondent answered a supplementary question if this longstanding illness causes a reduced work capacity or limits one’s other daily activities to a great extent, to some extent or not at all. LLSI was defined as having a longstanding illness that limited the work capacity or other activities to at least some extent. *Mental health* status was assessed using the General Health Questionnaire 12 (GHQ-12) which was included in the survey [39]. The 12 items of the GHQ-12 measure interruptions to normal function rather than life-long characteristics and are designed to capture mental reactions to strains. Each question is scored 0 for good mental well-being or 1 for poor, giving a maximum of 12 points and a minimum of 0. A summary score of three and above was considered as decreased mental wellbeing. It has been validated for use in the Swedish population and a score of three and above is used in Sweden to denote significantly decreased mental wellbeing [40]. *Somatic disease* was a combined measure including five diseases available in the survey questions about whether the respondent ever had been diagnosed for any of these by a physician: diabetes, angina pectoris, myocardial infarction, heart failure or cerebral haemorrhage. *Musculoskeletal pain* was created by combining three survey questions regarding the presence of pain in the neck or upper back, the lower back and shoulders or arms during the previous six months. Respondents who reported pain in either of these body parts “a couple of days a month” or more often were considered as having musculoskeletal pain. The sixth measure was *in-patient care* during the year of exposure (2002) based on information from the National Patient Registry.

### Statistical analyses

Descriptive analyses of background factors and potential confounders were computed from frequencies, and the differences were tested with Chi-square tests with associated p-values.

The odds ratios (OR) with corresponding 95% confidence intervals (CI) were obtained using logistic regression in order to model the effect of STSL on the risk of future long-term sick leave, disability pension, and short- and long-term unemployment. In the analyses, the effect was adjusted for the confounders in eight multivariate regression models.

Variables derived from the population-based registers did not yield any partially missing values. In the surveys there were some partially missing answers, ranging from 0.3% for country of birth to 1.7% for socio-economic status; hence the number of individuals included in the different models differs slightly. Excluding all individuals with partially missing answers (n= 550, 5.6%), basing the analyses solely on those with complete information on all variables, did not alter the effect estimates.

All statistical analyses were performed using the SAS statistical software, version 9.2 (SAS Institute Inc., Cary, NC, USA).

## Results

A total of 31.8% of the participants, reported low STSL during the last 12 months in 2002, and 18.5% reported high STSL during the same period (Table 1). There were statistically significant differences between sex and age groups in the prevalence of STSL. Among women 22.4% reported high STSL, compared to 14.6% among men, and among 18–29 year olds, 34.3% reported high STSL compared to 21.4% and 11.3% in the older age groups. Prevalence differences were also detected between socioeconomic groups, 8.0% of self-employed individuals reported high STSL compared to 18-22% among other socio-economic groups. Among those with less than good self-rated health, 27.3% reported high STSL, compared to 17.0% among the others. Similarly, 26.7% of individuals with decreased mental wellbeing reported high STSL, compared to 16.7% among those with good mental wellbeing.

In Table 2, differences in the number and percentage of the outcomes 2006–2007 is shown, stratified on STSL. Among those exposed to high STSL, 8.8% had long-term sick leave in 2007, compared to 4.6% among those with no STSL. There were similar differences between exposure groups in the prevalence of short-term unemployment in 2006, although the general prevalence figures were slightly lower. There were no statistically significant differences in the prevalence of disability pension in 2007 or long-term unemployment in 2006 between the exposure groups.

**Table 2:** Short-term sick leave (STSL) in 2002, number and percentage of the outcomes, in 2006–2007

**Short term sick leave (STSL) in 2002**	**Outcome measures**											
	**Sick leave more than 30 days in 2007**			**Disability pension in 2007**			**Unemployment in 2006 ≤180 days**			**Unemployment in 2006 >180 days**		
	**n**	**%**	p-value*	**n**	**%**	p-value*	**n**	**%**	p-value*	**n**	**%**	p-value*
High STSL (> 1 spell) n=1824	160	8.8	<0.0001	17	0.9	0.2377	94	5.2	<.0001	11	0.6	0.1063
Low STSL (1 spell) n=3125	197	6.3		29	0.9		103	3.3		38	1.2	
No STSL n=4895	225	4.6		63	1.3		146	3.0		54	1.1	
Missing	0			0			127			0		

*P-value for Chi-square tests.

Table 3 shows statistically significant higher crude odds ratios for long-term sick leave and short-term unemployment among those exposed to high STSL, and a significant higher odds ratio for long-term sick leave for those exposed to low STSL. No increased odds ratios were found for short-term unemployment for individuals exposed to low STSL. No increased odds ratios were found for long-term unemployment and disability pension. Overall, adjustment for all measures of health status in 2002 and socio-demographic factors only slightly changed the estimates. After these adjustments the odds ratio of future long-term sickness absence for those with high STSL was 2.11 (95% CI 1.67-2.67). The odds of long-term sick leave appeared to increase with increasing exposure. There was a crude increased odds of short-term unemployment among those exposed to high STSL, but after adjustment for mental wellbeing and socio-demographic factors, the effect estimates were not statistically significant (OR 1.25 95% CI 0.93-1.67). After adjustments for confounding no statistically significant effect of STSL was found on either long-term unemployment (OR 0.56 (95% 0.26-1.18) or disability pension (OR 0.74 95% 0.42-1.33).

**Table 3:** The association between short-term sick leave and long-term sick leave, disability pension and unemployment

**Regression model**		**Sick leave >30 days in 2007 (n=582)**	**Disability pension in 2007 (n=109)**	**Unemployment in 2006 ≤180 days (n=343)**	**Unemployment in 2006 >180 days (n=103)**
	**Short-term sick-leave spells (STSL)**	**OR (95% CI)**	**OR (95% CI)**	**OR (95% CI)**	**OR (95% CI)**
Crude	No STSL	1.00	1.00	1.00	1.00
	Low STSL	1.40 (1.15-1.70)	0.72 (0.46-1.12)	1.10 (0.86-1.43)	1.10 (0.73-1.68)
	High STSL	2.00 (1.62-2.46)	0.72 (0.42-1.24)	1.76 (1.35-2.29)	0.54 (0.28-1.04)
Model 1 (M1): Short- term sick leave adjusted for socio-demographic factors	No STSL	1.00	1.00	1.00	1.00
	Low STSL	1.48 (1.21-1.81)	0.81 (0.51-1.28)	1.01 (0.77-1.31)	1.34 (0.86-2.07)
	High STSL	2.27 (1.81-2.84)	1.08 (0.62-1.90)	1.33 (1.00-1.76)	0.70 (0.35-1.42)
Model 2: M1 and self-rated health (SRH)	No STSL	1.00	1.00	1.00	1.00
	Low STSL	1.47 (1.20-1.81)	0.75 (0.47-1.19)	1.01 (0.78-1.32)	1.32 (0.85-2.06)
	High STSL	2.16 (1.72-2.72)	0.85 (0.48-1.50)	1.34 (1.01-1.78)	0.65 (0.32-1.33)
Model 3: M1 and limiting longstanding illness (LLSI)	No STSL	1.00	1.00	1.00	1.00
	Low STSL	1.48 (1.21-1.82)	0.81 (0.51-1.29)	0.99 (0.76-1.29)	1.34 (0.86-2.07)
	High STSL	2.16 (1.72-2.71)	0.93 (0.53-1.64)	1.32 (0.99-1.75)	0.68 (0.33-1.37)
Model 4: M1 and mental wellbeing (GHQ12)	No STSL	1.00	1.00	1.00	1.00
	Low STSL	1.51 (1.22-1.85)	0.78 (0.49-1.23)	1.01 (0.77-1.31)	1.33 (0.86-2.08)
	High STSL	2.29 (1.82-2.87)	1.02 (0.58-1.80)	1.25 (0.93-1.67)	0.60 (0.29-1.25)
Model 5: M1 and musculoskeletal pain	No STSL	1.00	1.00	1.00	1.00
	Low STSL	1.47 (1.19-1.80)	0.76 (0.48-1.21)	1.01 (0.77-1.31)	1.31 (0.84-2.03)
	High STSL	2.18 (1.74-2.73)	0.94 (0.54-1.65)	1.34 (1.01-1.78)	0.66 (0.33-1.34)
Model 5: M1 and somatic disease	No STSL	1.00	1.00	1.00	1.00
	Low STSL	1.47 (1.20-1.80)	0.81 (0.52-1.29)	0.99 (0.76-1.29)	1.37 (0.88-2.13)
	High STSL	2.24 (1.78-2.81)	1.07 (0.61-1.88)	1.34 (1.01-1.78)	0.72 (0.36-1.46)
Model 7: M1 and in-patient care in 2002	No STSL	1.00	1.00	1.00	1.00
	Low STSL	1.47 (1.20-1.80)	0.78 (0.50-1.24)	1.00 (0.77-1.30)	1.35 (0.87-2.09)
	High STSL	2.26 (1.80-2.83)	1.07 (0.61-1.87)	1.32 (1.00-1.76)	0.71 (0.35-1.43)
Model 8: M1 and GHQ12, SRH, LLSI, musculoskeletal pain, somatic disease, in-patient care in 2002	No STSL	1.00	1.00	1.00	1.00
	Low STSL	1.49 (1.21-1.85)	0.73 (0.45-1.18)	0.98 (0.75-1.29)	1.37 (0.87-2.14)
	High STSL	2.11 (1.67-2.67)	0.74 (0.42-1.33)	1.29 (0.97-1.74)	0.56 (0.26-1.18)

Odds ratios (OR) of the outcomes, (with 95% confidence interval (CI)) among individuals with high (> one sick-leave spell), low (one sick-leave spell) and no short-term sick leave (STSL). Crude OR are shown, followed by seven multivariate models adjusted for health status and socio-demographic factors.

## Discussion

Having high STSL in 2002 increased the odds of future long-term sick leave in 2007. The increased odds of long-term sick leave remained after adjustment for socio-demographic factors and self-reported health status and register data on in-patient care from 2002. The results did not support STSL (high or low) to be a substantial risk factor for adverse labor market position. Exposure to STSL implied no statistically significant increased risks of unemployment or disability pension after adjustment for health status and socio-demographic factors.

Studies on the consequences of short-term sickness absence are scarce; however, our results are in line with those published [11,18]. These studies showed that frequent short-term absence was a predictor for later long-term sickness absence [18] and that sickness absence of more than 15 days per year was a risk factor for terminated employment, mainly resulting in unemployment [11,29]. In our study, we found an increased risk of long-term sick leave among individuals with STSL. The OR of short-term unemployment was above one but not statistically significant after adjustment for all measures of health status. Few of these previous studies had the possibility to control for health status. The differences between our study and previous studies, regarding the risk of unemployment, could be due to the differences in exposure definitions or due to the fact that the association previously found between sick leave and unemployment mainly is due to the ill health that sick leave reflects.

Another study showed that the duration of sick-leave spells and the total number of sick-leave days in a year were the strongest predictors of disability pension, which led the researchers to conclude that the pathway to disability pension starts with short term sick-leave periods, then increasing in length until the disability pension [31]. Our findings show that STSL increased the odds of long-term sick leave, but not of disability pension. However, it is important to note that our study investigated an initially healthy cohort, and it is likely that it may take longer than the five-year follow up to transition from short-term sickness absentee, to long-term sickness absentee to disability pensioner. The time period from short-term to long-term sick leave and later exclusion from the labor market may depend on severity of disease and type of work load but we have not found any studies regarding this issue.

### Strengths and limitations

The strength of this study is firstly the longitudinal design and the large number of participants, and secondly the opportunity to combine survey data and register data, available from 2001 until 2007. This allowed us to limit the sample by excluding persons with long-term sick leave and in-patient care in 2001 and thereby avoid short-term STSL in 2002 being a continuation of previous severe illness [41].

In order examine the consequences of sick leave when controlling for health status, we used our access to self-reported health assessments in the survey from 2002. This captured different aspects of general health measured by SRH, GHQ-12, and LLSI. The GHQ-12 questionnaire is a well-established and validated instrument, measuring mental well-being [41]. Further, the questions covering long-term limiting illness and musculoskeletal pain capture the most common somatic diagnosis among sickness absent, e.g. musculoskeletal diseases. The measure somatic disease covered severe diagnoses such as myocardial infarction or diabetes, but is not an all-encompassing measure of somatic diagnoses, however the access to registered in-patient care made it possible to control for those with ill health that required in-patient care for shorter or longer periods during 2002, like tumors, psychiatric diseases, and coronary heart diseases. Hence, the analyses were adjusted for both self-reported and register data on diseases and health conditions. Nevertheless, it is not possible to fully discriminate between the consequences of sick leave and the ill-health as we cannot link the health status with each specific sick-leave spell. Despite this shortcoming, we believe that this study has a major advantage compared to several previous studies of the consequences of sick leave [32,33], in its adjustments for both self-reported and registered ill health.

Previous studies indicate that adverse work conditions and smoking are risk factors for disability pension [25-28]. Since these risk factors are likely to have their effect on disability pension through illness and sick leave, we have not considered them as confounders in our study.

One weakness in our study is the lack of register data on exposure, which implies an inexact exposure measurement and may imply recall bias and risk of response bias due to social desirability. The registers of sick leave in Sweden do not have valid data on the first 14 days of sick leave, since the 14 first days of sick leave are compensated by the employer and hence not included in the register held by the Swedish National Social Insurance Agency. Data on exposure to STSL were thus based on self-reported survey data. However, studies on the validity and reliability of register data and self-reported data on sick leave have not shown any significant differences between these two types of information [42-44]. The lack of register data also implies that the outcome long-term sick leave will only include individuals with sick-leave spells of at least 15 days, since these are the ones registered by the Swedish National Social Insurance Agency. Unfortunately, no other register data on the outcome long-term sick leave is available. If differential, this misclassification is likely to be more common among the exposed group, which would underestimate the odds ratios of long-term sick leave.

STSL can been seen as a coping strategy to prevent later long-term sick leave [45], or as an indicator of an underlying severe disease [41]. In this study, those with chronic or severe disease, recognized by long-term sick leave, in-patient care, or disability pension the year before the short-term absence, were excluded from the study group. Hence, the study population was a “healthy population”, and most likely their subsequent ill health was either of short-lasting nature, or the beginning of a potential chronic, but not yet medically diagnosed, health condition. However, as mentioned above, to successfully investigate if a pathway exists from STSL, via long-term sick leave, to long-term labor-market exclusion, a longer follow up period than five years is likely to be needed. From our results we cannot determine through which mechanisms STSL affect long-term sick leave. Possible mediators between short-term and long-term sick leave remain for future studies to explore.

## Conclusions

Short-term sick leave increased the odds ratio for future long-term sick leave, when adjusting for several measures of health status. This may imply that short-term sick leave has social and health-related consequences beyond the effects of the underlying ill health it reflects. The results point to the importance for workplaces to pay attention to employees’ short-term sick leave in order to prevent subsequent long-term sick leave and to work with employee groups with high short-term sick leave in order to explore ways to reduce possible negative consequences. The results, however, showed no increased risk for disability pension or for longer unemployment.

## Competing interests

The authors declare no competing interests.

## Authors’ contributions

JM and CL conceived the study idea. HH and MM performed the statistical analyses. All authors participated in the design and coordination of the study, the interpretation of the results, and drafted the manuscript. All authors read and approved the final manuscript.

## Pre-publication history

The pre-publication history for this paper can be accessed here:

http://www.biomedcentral.com/1471-2458/12/861/prepub
